# Supplementary material for: The molecular architecture of tunneling nanotubes
Source: bioRxiv. 2026 May 28:2026.05.27.728322. Preprint. [Version 1] doi: 10.64898/2026.05.27.728322 (PMC13232226; doi:10.64898/2026.05.27.728322)
Supplement: 5 [file NIHPP2026.05.27.728322v1-supplement-5.pdf]

bioRxiv preprint doi: https://doi.org/10.64898/2026.05.27.728322; this version posted May 28, 2026. The copyright holder for this preprint (which was not certified by peer review) is the author/funder, who has granted bioRxiv a license to display the preprint in perpetuity. It is made available under a CC-BY 4.0 International license.

Supplementary Table 1: Cryo-ET data collection and model parameters

	#1 A549-US3<br>(EMDB-xxxxx: microtubule, pf-12)<br>(EMDB-xxxxx: microtubule, pf-13)<br>(EMDB-xxxxx: ribosome)	#2 THP1<br>(EMDB-xxxxx: microtubule, pf-13)
	**Data collection and processing**	
Magnification	53k	
Voltage (kV)	300	
Total dose (e–/Å^2)	160	
Defocus range (µm)	-3.0 to -5.0	
Defocus increment	0.5	
Acquisition scheme	Dose-Symmetric, - 37.5/37.5, 2.5° step, group 2	
Pixel size (Å)	1.635	
No. of frames	8	
# of tomograms	168	38
# of subtomograms	29,514 (microtubules)<br>29,792(ribosomes)	2,920
Final particle #	10,653(microtubule, pf-13)<br>3,183(microtubule, pf-12)<br>2,402(ribosomes)	2,920
Symmetry imposed	C1 for ribosomes,<br>-27.7° rot and 9.4 Å rise for pf-13 microtubule<br>-29.88° rot and 10.4 Å rise for pf-12 microtubule	
Map resolution (Å)<br>FSC threshold = 0.143 (Å)	13.3 (microtubule, pf-12),<br>8.0 (microtubule, pf-13),<br>9.1 (ribosome)	8.7 (microtubule, pf-13),
Map resolution range (Å)	8 to >14	8.7 to >9.5
	**Fitting**	
Model used (PDB code)	1TUB, 5LZS	1TUB

Supplementary Table 1. Cryo-ET data collection, processing, and map parameters.
